# Symptomatic seizures in preterm newborns: a review on clinical features and prognosis

**DOI:** 10.1186/s13052-018-0573-y

**Published:** 2018-11-01

**Authors:** Carlotta Spagnoli, Raffaele Falsaperla, Michela Deolmi, Giovanni Corsello, Francesco Pisani

**Affiliations:** 1 Child Neuropsychiatry Unit, Department of Pediatrics, Arcispedale Santa Maria Nuova, IRCSS, Reggio Emilia, Italy; 2 Neonatal Intensive Care Unit, Santo Bambino Hospital, University Hospital “Policlinico-Vittorio Emanuele”, Via Tindaro 2, 95124 Catania, Italy; 3 Pediatrics Unit, Medicine & Surgery Department, University of Parma, Parma, Italy; 4 Department of Maternal and Child Health, University of Palermo, Palermo, Italy; 5 Child Neuropsychiatry Unit, Medicine & Surgery Department, Neuroscience Division, University of Parma, Parma, Italy

**Keywords:** Seizures, Newborn, Outcome, Prognosis, Treatment

## Abstract

Neonatal seizures are the most common neurological event in newborns, showing higher prevalence in preterm than in full-term infants. In the majority of cases they represent acute symptomatic phenomena, the main etiologies being intraventricular haemorrhage, hypoxic-ischemic encephalopathy, central nervous system infections and transient metabolic derangements.

Current definition of neonatal seizures requires detection of paroxysmal EEG-changes, and in preterm newborns the incidence of electrographic-only seizures seems to be particularly high, further stressing the crucial role of electroencephalogram monitoring in this population. Imaging work-up includes an integration of serial cranial ultrasound and brain magnetic resonance at term-equivalent age. Unfavourable outcomes following seizures in preterm infants include death, neurodevelopmental impairment, epilepsy, cerebral palsy, hearing and visual impairment. As experimental evidence suggests a detrimental role of seizures per se in determining subsequent outcome, they should be promptly treated with the aim to reduce seizure burden and long-term disabilities. However, neonatal seizures show low response to conventional anticonvulsant drugs, and this is even more evident in preterm newborns, due to intrinsic developmental factors. As a consequence, as literature does not provide any specific guidelines, due to the lack of robust evidence, off-label medications are often administered in clinical practice.

## Background

Owing to improved neonatal care strategies and implemented medical technology, the number of surviving preterm newborns is increasing, even among extremely low birth weight babies, although their morbidity rate is higher compared to full-term infants [1]. Hence, new challenges for the medical community include the management and treatment of clinical conditions more often affecting preterm infants, including neonatal seizures, which are the most common neurological event in newborns. In fact, there are several characteristics of preterm babies, such as immature central nervous system development, low birth weight and periventricular leukomalacia, that also represent risk factors for seizures [2]. However, preterm newborns are also more prone to develop severe complications and they are less responsive to treatment with conventional medications because of age-specific pharmacokinetic and pharmacodynamic features [3]. In spite of these differences between preterm and full-term infants, there is no difference in preterm versus full-term treatment strategies in clinical practice, even if literature data suggest that the same treatment might be less effective and more dangerous in preterm than in full-term newborns. As a consequence, second-line or off-label medications use has been increasingly reported. Irrespective of controversies in their management, neonatal seizures treatment is considered very important in order to reduce morbidity, mortality and seizure burden, and to improve the overall outcome in these patients.

## Epidemiology

Neonatal seizures affect up to 1.5–3.5/1000 full-term newborns and 10–130/1000 preterm infants [4]. Therefore, the prevalence of seizures in preterm newborns is higher than in full-term ones (22.2% compared to 0.5%) [5], even if a recent study found that the incidence of seizures is lower between 30 and 36 weeks of gestational age than in both extremely preterm infants (<30w GA) and in near-term ones (36-40w GA) [6].

Furthermore, time of seizure onset seems to be different between preterm and full-term newborns: in fact, they tend to occur later in premature infants. In particular, the first suspected seizure is reported at a median age of 27 h of life in full-term infants and at 14 days in preterm newborns [7]. Moreover, there are some differences according to gestational age: in a recent study mean seizure onset was 14.6 days (range 1–120 days, SD 20.4) in <29wGA newborns, compared to 5.2 days (range 1–37 days, SD 7.7) in >29wGA ones [8]. In a further research, mean seizure onset was 8.3 days of life in infants with gestational age ≤ 29 weeks, while it was earlier (3.2 days) in newborns with a gestational age between 30 and 36 weeks [9]. This is likely due to differing seizure etiologies [6, 8–10]. In particular, seizures caused by intraventricular haemorrhage usually occur during the first 24 h of life and many preterm newborns’ seizures persist into the third day of life [11]. Especially if associated with parenchymal infarction, seizures are most likely to occur in the latter part of the first week of life. Other etiology of seizure in preterm are represented by hypoxic-ischemic encephalopathy, central nervous system infections and transient metabolic derangements [12].

Moreover, the rate of seizures seems to depend on the method of ascertainment, as the prevalence of clinically-defined seizures ranges from 0,30 [13] to 57.5 per 1000 births [14] while the prevalence of EEG-detected seizures is 24% in premature infants [10].

## Etiology

Intraventricular haemorrhage and its complications are the main cause of neonatal seizures in very and extremely preterm newborns (< 32 wGA) [8, 10], and the risk increases with increasing severity of brain injury (grades III and IV intraventricular haemorrhage) [8, 15–17]. On the other hand, in moderate and late preterm infants the majority of seizures are caused by hypoxic-ischemic encephalopathy [10] which shows 40–60% prevalence in near-term newborns with seizures [17, 18]. Additional acquired etiologies include central nervous system or systemic infection (i.e. meningitis, viral meningoencephalitis and sepsis) [8], other acquired brain injuries (subarachnoid haemorrhage, ischemic stroke), trauma, transient metabolic diseases (i.e. hypoglycaemia in low birth weight infants, hypocalcaemia, hypomagnesaemia, sodium imbalance in very low birth weight ones) [12], drug withdrawal or poisoning [20]. Neonatal-onset epilepsies (of genetic origin) and inborn errors of metabolism also need to be considered in the differential diagnosis.

## Pathophysiology

Various risk factors have been associated with seizures in preterm newborns, such as lack of state changes on EEG [21], intraventricular haemorrhage, periventricular leukomalacia, surgery, patent ductus arteriosus, necrotizing enterocolitis, pulmonary diseases or respiratory distress and low birth weight [19]. In particular, low birth weight infants show a 9% increase in seizure rate for each week decrease in gestational age [19].

However, the main reason why preterm infants are prone to develop seizures is their brain immaturity [22].

Gamma-Aminobutyric acid (GABA) is crucial for neuronal maturation and activity-dependent integration into circuits [23]. Immature neurons arecharacterized by high neuronal chloride concentrations, deriving from the different expression of the NKCC1 versus the KCC2 cation-chloride cotransporters in immature versus mature neurons [24, 25]. This causes GABAergic signaling to be mainly excitatory and driven by NKCC1 receptors in embryonic and early post-natal life [26]. Afterwards, the GABAergic interneuronal network develops and switches from excitatory to inhibitory. Neurophysiologically, there is a concomitant emergence of more ‘continuous’ oscillations subsiding the spontaneous activity transients (SATs), recorded in extremely preterm newborns [27].

Between 24 and 32 weeks of gestational age, premyelinating oligodendrocyte progenitor cells are particularly vulnerable to hypoxic-ischemic damage [28], due to the interaction of immature blood flow autoregulation, immature vascular supply and heart-rate dependent cardiac output [22, 29]. Consequently, white matter injury occurs when other predisposing factors such as infections, inflammations and disturbances in cerebral oxygenation overlap with the intrinsic vulnerability of the premature brain [30]. Oligodendroglial damage causes impaired myelination, while parenchymal haemorrhage or high grade intraventricular haemorrhage cause projection and association fibers injury. When changes affect subplate neurons, both the cortex and its connections with the thalamus are damaged [28], and this can lead to higher long-term cortical excitability [31].

In particular, extremely low birth weight newborns often display a passive cerebral blood flow [32]. As a consequence, the occurrence of intraventricular haemorrhage is more frequent, and various hypotheses have been proposed to explain this higher risk. First of all, some conditions such as pneumothorax, changes in head position and ventilator asynchrony can cause impaired venous drainage, thus facilitating haemorrhage. Second, sudden changes in arterial blood pressure caused by sepsis, noxious stimuli, fluid boluses or inotropes can have the same effect, leading to intraventricular haemorrhage [29].

## Diagnosis

Neonatal seizures in newborns are clinically defined as abnormal, stereotyped and paroxysmal dysfunctions in the central nervous system, occurring within 44 weeks of gestational age.

From a clinical point of view, neonatal seizures are divided into clonic, myoclonic, tonic and subtle seizures. Clonic seizures consist of repetitive, rhythmic (1–4/s) contractions of muscle groups of the limbs, face or trunk. They can be focal or multifocal. They are usually electrically-confirmed. Myoclonic seizures are isolated or repetitive contractions of proximal or distal muscles, less regular and persistent than clonic jerks. They can be generalized, focal or multifocal. Tonic seizures are classified as generalized or focal. Focal tonic seizures consist of sustained, but transient, asymmetrical posturing of the trunk or extremities or tonic eye deviation, while generalized tonic seizures are bilateral symmetrical tonic posturing (with flexor/extensor predominance or mixed). Electrographic correlate is inconsistent for both seizure types. Subtle seizures include motor automatisms (such as chewing, swallowing, sucking, repetitive tongue movements, “cycling”, “boxing”, “pedaling”, “swimming”) and autonomic signs (changes in heart rate or breathing pattern, flushing, salivation, pupil dilatation) [33].

Neurophysiologically a seizure is defined as the presence of a paroxysmal EEG change with a clear onset and termination, lasting at least 10 s, and an evolution in frequency, morphology and amplitude.

As above said, the prevalence of neonatal seizures is higher in preterm (22.2%) than in full-term newborns (0.5%) [5] but studies about clinically-defined seizures show a larger percentage of neonatal seizures than EEG-based studies [34, 35], especially aEEG-based ones [11, 15]. For example, a prospective study about infants born before the 32nd week of gestational age found a 5% incidence of neonatal seizures [36]. In contrast, 11.9% incidence has been reported in studies on clinically-diagnosed seizures [6].

Clinical-only seizures (without any electrographic evidence), are usually considered non-epileptic and related to the loss of cortical inhibition on subcortical structures [37].

Paroxysmal non-epileptic motor phenomena should be taken into consideration in the differential diagnosis [38].

No general consensus has been achieved on the definition of neonatal status epilepticus. A commonly adopted definition considers it as continuous seizure activity lasting ≥30 min or the occurrence of recurrent seizures ≥50% of the recording time (1–3 h) [18]. For a recent review on controversies regarding status epilepticus, please refer to (Pavlidis, Pisani EJPN 2018) [39].

## Monitoring of preterm newborns at increased risk of seizures

If possible, a multimodal neurophysiological and neuroimaging approach is recommended in newborns [40].

### Neurophysiologic monitoring: Conventional and amplitude-integrated EEG

Literature points out the importance of EEG monitoring in the context of suspected seizures, both for diagnosis and to evaluate treatment efficacy.

Conventional video-EEG enables definition of seizure onset, spread, duration and correlation with motor phenomena. Therefore, it can differentiate paroxysmal non-epileptic motor phenomena (not associated with any EEG changes) and detect electrographic-only seizures [41]. It can give early prognostic information, suggesting etiologies such as a focal injury or a diffuse pathology and indicates severity of functional compromise [34, 38, 42, 43]. In fact, background activity is a strong predictor of outcome, in particular when serial recordings are performed [44]. Moreover, even after seizure confirmation, prolonged or continuous monitoring are useful to quantify “seizure burden” and to diagnose neonatal status epilepticus [45, 46]. EEG-monitoring is required to test anticonvulsants efficacy, so it is recommended to continue EEG-recording for at least 24 h of seizure freedom [47], and it is also advisable to monitor the newborn after drug discontinuation. In addition, the literature suggests that amplitude-integrated EEG (aEEG) might miss seizures originating far from the electrodes or of short duration. It can also lead to false positive results due to artefacts. Consequently, it is mainly used as a screening tool, while conventional EEG is recorded to improve sensitivity and specificity [48, 49]. In preterm newborns, the first EEG-recording is recommended as soon as possible after birth, because its sensitivity is higher in the first 48 h of life [50, 51].

### Cranial ultrasound scan

Another non-invasive and low cost technique to detect seizure etiology is cranial ultrasound scan (CUS). Therefore, a scan protocol for preterm infants has been proposed. In particular, a CUS is always recommended at term equivalent age in preterm newborns and, from the 23rd to the 35th week of GA, a CUS should be performed in the first 24 h of life, then at the first, second and third week of life. Additional cranial ultrasound scans are recommended depending on gestational age [52]. Moreover, CUS should be integrated with brain magnetic resonance (MRI) at term equivalent age when intraventricular haemorrhage, to detect ventriculomegaly, white matter injury and cerebellar involvement [53, 54].

### Brain MRI

First of all, brain magnetic resonance imaging (MRI) can recognize specific patterns in seizures due to structural causes, for example related to central nervous system infections [55]. Conventional brain MRI should be performed in preterm newborns at term-equivalent age, to gain more accurate information about ventriculomegaly, white matter injury and cerebellar involvement in case of uncomplicated intraventricular haemorrhage [53, 54]. In contrast, when hypoxic-ischemic encephalopathy is the seizures cause, brain injury can be detected earlier with diffusion weighted imaging (DWI), while conventional brain MRI shows a loss of grey-white matter differentiation at the end of the first week of life [56].

## Treatment

According to experimental and clinical data [57], prolonged seizures may contribute to poor neurological outcome, thus they should be promptly treated in an attempt to reduce long-term disabilities [58]. However, therapeutic options for seizures in newborns are still unsatisfactory [59] and the available evidence is poor [60].

Moreover, the literature does not provide any customized treatment approach for premature newborns suffering from neonatal seizures, even if several studies show a lower response rate than in full-term infants, with poorer prognosis. In fact, liver and renal function, but also central nervous system development, are not complete in premature infants, leading to differences in drugs pharmacokinetics and pharmacodynamics. Hence, potentially the same treatment might be both less effective and more dangerous in a preterm newborn than in a full-term one. However, studies found no difference in treatment strategies according to gestational age in clinical practice [61]. Conventional GABAergic antiseizure medications are less effective in newborns, because GABAergic signalling is mainly driven by NKCC1 receptors, with an excitatory instead of inhibitory effect [24, 26]. As a consequence, second-line or off-label medications are increasingly administered to newborns and proposed in treatment algorithms, although the underlying evidence is low [62]. The recommended first-line choice is represented by phenobarbital in both term and preterm infants (80% of cases), even if its efficacy is approximately 50%, followed by phenytoin in 40% of patients. This is based on the availability of randomized controlled safety and efficacy data [63]. In the lack of clear-cut evidence or guidance, when no response is obtained with conventional antiseizure drugs, off-label agents are often administered, such as levetiracetam, topiramate, lidocaine and midazolam. [3, 62, 64, 65]. Studies about pharmacokinetics and pharmacodynamic changes in preterm infants, report a longer half-life and a higher distribution volumes in preterm than in full-term newborns for both first-line and second-line antiseizure medications [66–69]. Anyway, it is important to notice that in recent years, levetiracetam has been increasingly used for treatment of neonatal seizures. Along with other papers [64, 70–73, 74], a recent retrospective analysis on preterm infants treated with levetiracetam as first-line showed 57% of seizure freedom at the end of the first week of life, without requiring any other treatment, no adverse drug reactions or laboratory abnormalities, suggesting that levetiracetam can be a safe option in neonates [75].

Finally, no clear evidence on when to discontinue medications is available, although in the majority of newborns therapy is continued after discharge from neonatal units [10]. A recent perspective research demonstrates that in neonates suffering from hypoxic-ischemic encephalopathy, antiseizure medication discontinuation prior to discharge did not increase the risk of seizures after discharge [75]. A further recent study suggests to use the need for a second antiseizure medication and time to seizure control to determine when to discontinue phenobarbital, supporting its early discontinuation [76].

## Outcome and risk factors for unfavourable outcome

Seizures in preterm newborns result in an increased risk of developing several medical conditions with neurological and intellectual disabilities. Recent papers show that, thanks to innovations in neonatal care, there has been a reduction in mortality, but, as a consequence, an increase in morbidity [77, 78]. In fact, common unfavourable outcomes include epilepsy, cerebral palsy [77], cognitive impairment (intellectual disability and developmental delay, 64% vs 29% in preterm infants with seizures compared to those without seizures), microcephaly [79], hearing (11% vs 4% in preterm newborns without seizures) and vision impairment (43% vs 14%).

Studies detect a normal long-term outcome in a small percentage of patients (12–25%). In comparison, a normal outcome is more common in both full-term infants suffering from seizures [18, 78, 80] and in preterm infants without seizures [81].

In accordance with these data, mortality rate is higher in preterm (32–35%) than in full-term newborns with seizures (5.4–15%) [10, 80, 82] and in preterm infants without seizures (5–16.6%) [35, 82]. Risk factors for mortality include birth weight lower than 1000 g, birth at GA < 28 weeks and severely abnormal background EEG patterns, such as isoelectric or low voltage invariant activity, burst-suppression or permanent discontinuous activity, and longer seizure duration, namely status epilepticus [8, 9]. In the context of status epilepticus, the mortality risk is higher in preterm compared to full-term newborns (52,6% versus 17,8%), even though, interestingly no statistically significant differences between preterm and full-term infants with status epilepticus were documented, except for Apgar scores and neurological examination [46].

Notably, background EEG pattern represents an independent predictor of mortality [8]. When seizures are exclusively subclinical, mortality rate is higher [7], while late death (after 36 weeks of post-menstrual age) and neuro-developmental impairment have been reported as more frequent in patients with clinical seizures [82].

Additional risk factors for poor prognosis include male sex, multiple birth, non-Caucasian newborns, low birth weight [82] and poor response to anticonvulsant drugs [7]. This latter factor has been associated with a higher rate of later epilepsy, together with severely abnormal cranial ultrasound scan and EEG (especially when the ictal activity spreads to the contralateral hemisphere) and status epilepticus [35, 43], as they underline more severe brain injury. In particular, status epilepticus is an independent predictor of unfavourable outcome, because it is associated with subsequent epilepsy in both preterm and full term infants [80]. Studies show that status epilepticus is more frequent in preterm infants with birth weight lower than 1000 g or between 1500 and 2499 g, with severely abnormal EEG traces and neurological examination [8, 46]. Anyway, it is important to notice that preterm newborns with status epilepticus show a lower incidence of post-neonatal epilepsy than full-term ones [18], even if still as high as 40 times higher than in the general population [78].

In contrast, cerebral palsy as an outcome in patients suffering from neonatal seizures seems to be higher in preterm infants than in full-term newborns [77], especially when prematurity is associated with periventricular leukomalacia, severe intraventricular haemorrhage, systemic disease or infections [83, 84]. Regarding the administration of steroids in newborns, there seems to be an increased risk of cerebral palsy when they are administered after birth, while the risk is lower if administered in the antenatal period [83].

Furthermore, with respect to intellectual impairment following neonatal seizures, evidence shows a high rate of severe impairment (61%), with lower percentage of moderate (22%) and mild (17%) impairment, without statistically significant differences associated to gestational age [9]. The risk of cognitive impairment seems to be higher in clinically-diagnosed than in EEG-diagnosed seizures [78, 81].

## Conclusion

Acute symptomatic neonatal seizures can be caused by several conditions which should be detected because prognosis largely depends on underlying etiology. The most frequent cause in preterm infants is intraventricular haemorrhage. Appropriate clinical and neurophysiologic monitoring, neuroimaging techniques and improvement in neurocritical care in the neonatal period are improving management of infants with seizures, and their outcome, especially mortality risk. However, the occurrence of seizures in preterm newborns is still associated with poor cognitive development, visual, hearing and motor impairment, cerebral palsy and epilepsy. Early treatment should be provided to neonates presenting seizures (even electrical seizures) to reduce mortality and disabilities and to improve long-term outcome especially in very and extremely preterm newborns.

## Future perspectives

In the future, research should be addressed to identify a customized treatment for preterm newborns, considering their pharmacokinetic and pharmacodynamic changes and brain developmental stages. Randomized controlled studies are required in order to establish if treatment strategies should be differentiated according to gestational age and underlying etiology (and how) with special emphasis on treatment duration and timing of treatment discontinuation.

## Abbreviations

aEEG: Amplitude-integrated electroencephalogram
CUS: Cranial ultrasound scan
DWI: Diffusion weighted imaging
EEG: Electroencephalogram
GA: Gestational age
GABA: Gamma-Aminobutyric acid
MRI: Magnetic resonance imaging
SAT: Spontaneous activity transient
SD: Standard deviation
